# Cerebrospinal Fluid Biomarkers for Alzheimer’s Disease in the Era of Disease-Modifying Treatments

**DOI:** 10.3390/brainsci11101258

**Published:** 2021-09-23

**Authors:** George P. Paraskevas, Elisabeth Kapaki

**Affiliations:** 1 2nd Department of Neurology, School of Medicine, National and Kapodistrian University of Athens, “Attikon” General University Hospital, 12462 Athens, Greece; 2 1st Department of Neurology, School of Medicine, National and Kapodistrian University of Athens, Neurochemistry and Biological Markers Unit, “Eginition” Hospital, 11528 Athens, Greece; ekapaki@med.uoa.gr

**Keywords:** Alzheimer’s disease, cerebrospinal fluid, biomarkers, beta amyloid, phospho-tau, aducanumab, anti-amyloid antibodies

## Abstract

Correct in vivo diagnosis of Alzheimer’s disease (AD) helps to avoid administration of disease-modifying treatments in non-AD patients, and allows the possible use of such treatments in clinically atypical AD patients. Cerebrospinal fluid (CSF) biomarkers offer a tool for AD diagnosis. A reduction in CSF β-amyloid (marker of amyloid plaque burden), although compatible with Alzheimer’s pathological change, may also be observed in other dementing disorders, including vascular cognitive disorders due to subcortical small-vessel disease, dementia with Lewy bodies and normal-pressure hydrocephalus. Thus, for the diagnosis of AD, an abnormal result of CSF β-amyloid may not be sufficient, and an increase in phospho-tau (marker of tangle pathology) is also required in order to confirm AD diagnosis in patients with a typical amnestic presentation and reveal underlying AD in patients with atypical or mixed and diagnostically confusing clinical presentations.

## 1. Introduction

Besides atrophy and neuronal as well as synaptic loss, there are two pathologic hallmarks of Alzheimer’s disease (AD): extracellular accumulation of amyloid beta peptide in the form of amyloid plaques and hyperphosphorylation of tau protein which polymerizes in the form of paired helical filaments, the key component of intraneuronal neurofibrillary tangles [1]. From the clinical point of view, AD has long been considered as synonymous with amnestic dementia of the hippocampal type [2], and in such typical cases, the accuracy of clinical diagnosis in specialized centers may approach or exceed 90% [3]. However, with time it became evident that AD may present with non-amnestic phenotypes [4], including the frontal type of dementia [5], posterior cortical atrophy [6], primary progressive aphasia [7] and even corticobasal syndrome [8]. Cases of AD mixed with cerebrovascular disease [9], Lewy body pathology [10] and normal-pressure hydrocephalus [11] are not uncommon, and these additional pathologies may affect the clinical presentation. Furthermore, some patients with AD may present at the pre-dementia stage of mild cognitive impairment (MCI) [12]. In such atypical or mixed cases, in early disease, in the community and in the presence of comorbidities, diagnostic accuracy decreases [13]. In ~1/3 of patients clinically diagnosed with AD, the final diagnosis may be different [14], whilst when a demented patient is considered not to suffer with AD, there is still a 39% chance of (co)occurrence of AD in an autopsy [15]. However, a correct diagnosis is needed in order to inform patients and relatives for the prognosis and for making the correct therapeutic decisions [16].

On June 2021, the anti-amyloid monoclonal antibody aducanumab [17] was approved by the Food and Drug Administration (FDA) in the USA [18] for the treatment of MCI or mild dementia due to AD [19]. The approval was based on the ability of the antibody to effectively remove brain amyloid, according to the initial PRIME study [17] (surrogate endpoint). From the clinical point of view, statistically significant cognitive benefits were observed in the high-dose branch of one study (EMERGE), while in a second study (ENGAGE) the results were not significant [20]. However, in a post hoc analysis of the latter study, data from patients receiving a high-dose treatment supported the positive findings of EMERGE [20]. Overall, the rate of cognitive decline seems to be reduced by ~23% [20], but questions concerning the drug’s efficacy and safety have been raised [21,22]. Recognizing this residual uncertainty, the FDA approved aducanumab under the accelerated approval pathway [18], in order to introduce a new treatment for such a devastating disease after more than 20 years. However, despite the discussion and the debates triggered [23], current recommendations suggest that if such a disease-modifying treatment is to be administered, the diagnosis of AD should not be based solely on clinical criteria, but a more objective tool, including the “signature pattern” or “profile” of AD, which cerebrospinal fluid (CSF) biomarker testing should confirm [24]. 

## 2. The CSF Alzheimer’s Disease Profile

During the last two decades, various CSF biomarkers have been developed for the (differential) diagnosis of AD [25,26]. With sensitivities and specificities approaching or exceeding 90%, they have been incorporated into various guidelines and diagnostic criteria for Alzheimer’s disease [12,27,28]. They include amyloid beta peptide with 42 amino acids (Aβ_42_), which is a marker of amyloid pathology, inversely correlated with the amyloid plaque burden [29], phospho-tau, mainly phosphorylated at a threonine residue at position 181 (τ_P-181_), which is a marker of tau protein hyperphosphorylation as well as tangle formation [30] and total tau protein (τ_T_), which is considered as a non-specific marker of neuronal/axonal degeneration [31]. Amyloid beta peptide with 40 amino acids (Aβ_40_) can also be measured in the CSF, and it is useful in the form of the Aβ_42_/Aβ_40_ ratio, which seems to perform diagnostically better than Aβ_42_ alone [32]. These biomarkers may be helpful in the recognition and/or differential diagnosis of AD in patients with a typical amnestic presentation as well as in cases with atypical or mixed presentations [33,34,35,36,37,38].

Initially it was suggested that the AD CSF biomarker profile is characterized by low Aβ_42_ (or low Aβ_42_/Aβ_40_) and an increase in either τ_P-181_ or τ_T_ [28]. Since typically in AD both τ_P-181_ and τ_T_ are increased, this “either” usually does not create diagnostic confusion. However, in the occasional patient with low Aβ_42_ (indicating the presence of amyloid plaque load) and increased τ_T_ (indicating neuronal/axonal damage of any cause), but normal τ_P-181_ (suggestive of absence of tangle formation), diagnostic doubt arises. Thus, it has been suggested that in addition to low Aβ_42_ both τ_P-181_ and τ_T_ should be increased in order to fulfill the AD profile [39].

**Figure 1:**
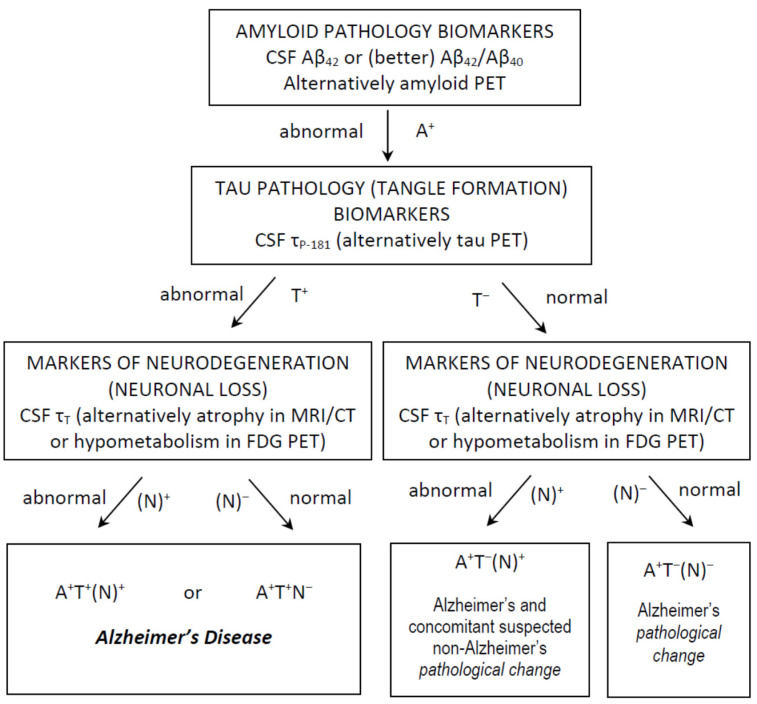
Use of CSF classical biomarkers and the AT(N) classification system in everyday clinical practice, for the diagnosis of Alzheimer’s *disease* and Alzheimer’s *pathological change*. Other profiles are not compatible with Alzheimer’s continuum and are not shown.

More recently, the so-called AT(N) system (A for amyloid load, T for tau accumulation and N for neurodegeneration) [40] has been proposed by the National Institute of Aging and Alzheimer’s Association (NIA–AA) Research Framework group, in which AD is viewed as a biological process, irrespective of the type (amnestic vs non-amnestic presentations), severity (MCI vs dementia) or even absence of symptoms (pre-clinical stage) at a certain time point [41]. In this context, the presence of reduced Aβ_42_ (or Aβ_42_/Aβ_40_) and increased τ_P-181_ is considered as compatible with an AD diagnosis, since this profile indicates the presence of both amyloid plaques and neurofibrillary tangles, whilst τ_T_ may be either increased or normal. The presence of atrophy in magnetic resonance imaging may also serve as a marker of nonspecific neurodegeneration, replacing τ_Τ_. Thus, a profile of A^+^T^+^(N^+^) or A^+^T^+^(N^−^) (^+^ for positive/abnormal test, ^−^ for negative/normal test) is compatible with AD [41] (Figure 1).

## 3. Other Profiles

In cases without amyloidosis in the CSF, presenting the A^−^T^+^(N*) profile (* for either ^+^ or ^−^), the AD diagnosis is not confirmed and this profile is usually compatible with one of the frontotemporal lobar degeneration pathologies (especially that of tau) [41]. On the other hand, the A^+^T^−^(N*) profile has extended differential diagnosis.

In Alzheimer’s disease, CSF biomarker changes may become evident 10‒20 years prior to the symptomatic stage [42,43,44]. Usually (but not always), the first biomarker to become abnormal is Aβ_42_, which is reduced early in the asymptomatic stage, followed by an increase in τ_P-181_, and it seems that most (but not all) AD patients enter the stage of MCI with both of the above biomarkers having become abnormal (although τ_P-181_ may not have reached the maximally abnormal level yet) [45,46]. Thus, in AD, A^+^T^−^(N^−^) is usually observed in the asymptomatic stage [47]. However, the biochemical/pathological and clinical progression of AD is gradual and sometimes overlapping, and some patients with the A^+^T^−^(N^−^) profile may experience subtle cognitive symptoms or even MCI [48]. This profile is considered extremely rare in the mild dementia stage of AD [45]. Thus, in AD the A^+^T^−^(N^−^) profile is practically always observed in the pre-dementia and, usually, at the preclinical stages; since neurodegenerative changes remain limited, atrophy and increased τ_Τ_ are usually not evident yet. The above profile in the AT(N) system belongs to the so-called pathological change in Alzheimer’s, and is considered as a part of the Alzheimer’s continuum, but not synonymous to AD [41].

On the other hand, the A^+^T^−^(N^+^) profile points to significant neurodegeneration and, since there is no indication of tangle formation, another (non-AD) pathology is suspected to be present together with amyloid pathology. A typical neurodegenerative disorder with this profile may be dementia with Lewy bodies (DLB) [38]. However, is the A^+^T^−^N* profile always indicative of pathological change in Alzheimer’s (amyloid plaques)? In subcortical small-vessel disease (SSVD), CSF amyloid reduction is not uncommon [9,37,49], and the same holds true for some patients with normal-pressure hydrocephalus (NPH) [50,51] and Creutzfeldt–Jakob disease [52], whilst amyloid pathology may be present in a small percentage of patients with corticobasal degeneration [53]. One could argue that a concomitant AD-type pathology is not uncommon in SSVD [54] and NPH [55]; however, low Aβ_42_ has also been observed in cerebral autosomal dominant arteriopathy with subcortical infarcts and leucoencephalopathy (CADASIL), where the co-occurrence of AD pathology is extremely rare [56]. Thus, the A^+^T^−^N* profile may not always be synonymous with amyloid pathology.

## 4. Limitations

There are some limitations in classical CSF biomarker determinations. Test results are affected by preanalytical factors, including CSF sampling and storage in addition to internationally accepted guidelines have been formulated for this reason [57]. In addition, international quality control programs have been organized in order to optimize analytical performance [58], and international projects have been launched with the aim of, among others, identifying and controlling for confounding factors, improving the methodologies used and harmonizing the levels of biomarkers [59]. Experience gained from such projects and strict adherence to guidelines may decrease measurement error [60]. However, there is still a significant intra- and inter-laboratory variability [61,62] and each laboratory should have its own cut-off values [63]. Diagnostically gray zones also exist and, if added to the possible measurement error, may lead to a variability of ±25%, especially important for the cut-off values [64].

Discordant biomarker results have been observed in different reference laboratories, especially for Aβ_42_, and various factors may be responsible, including the APOE ε4 allele [64]. The Aβ_42_/Aβ_40_ ratio, which performs better than Aβ_42_ alone [32,65] may reduce discordance to some extent [64], but there is still some concern about inconsistent results, especially in the AT(N) system [66].

Classical CSF biomarkers can identify the presence of AD in patients with mixed conditions, such as cerebrovascular disease (evident in neuroimaging) or DLB (fulfilling clinical criteria) with a concomitant AD pathology [9,36,37,38,54], but they cannot identify other neurodegenerative pathologies in the presence of AD, i.e., the A^+^T^+^(N)^+^ profile is simply compatible with the presence of AD, but gives no information for a possible additional synucleinopathy or, rarely, one of the frontotemporal pathologies. Furthermore, normal levels of all three CSF classical biomarkers may be observed not only in normal aging, but also in psychiatric disorders which may present with cognitive complaints [67,68] and in some of the frontotemporal pathologies [69], which may enter in the differential diagnosis of psychiatric disorders. Assessment of α-synuclein may be of value in the presence of Lewy body pathology [70], but results are conflicting [71,72]. Another emerging biomarker is TAR DNA-binding protein 43 (TDP43), which may prove helpful in identifying TDP43-related frontotemporal pathologies, especially when combined with the classical biomarkers [73]; much work still has to be done. The AT(N) system is flexible and may evolve into an ATX(N) system, incorporating new and emerging biomarkers of additional non-AD pathologies [74].

Finally, one must keep in mind that CSF biomarkers are not stand-alone tools. As in most (if not all) areas of medicine, biochemical tests should be interpreted along with clinical, imaging and other laboratory data (and sometimes follow-up data) in order to reach the final diagnosis. 

## 5. Concluding Remarks

According to the FDA approval, aducanumab targets parenchymal amyloid in Alzheimer’s disease and not in any condition with amyloid plaque burden (e.g., synucleinopathy with concomitant amyloid pathology). For CSF confirmation of an AD diagnosis, reduction in Aβ_42_ alone is not sufficient, since it may be compatible with pathological change in Alzheimer’s [41]; it may also be observed in other conditions such as DLB [38] or vascular cognitive impairment [54,56], which in some patients may present with oligosymptomatic phenotypes (especially early in the disease progress) and enter into the differential diagnosis of AD. Neuropathological diagnosis of AD requires both amyloid plaques and neurofibrillary tangles [75] and thus, for the in vivo diagnosis of AD by CSF biomarkers, both reduced Aβ_42_ (or Aβ_42_/Aβ_40_) and increased τ_P-181_ should be present [41]. Even if the AT(N) classification system [41], other guidelines/criteria [28,39] or scoring systems such as the “Erlangen score” [76] are not followed, it has been suggested that the τ_P-181_/Aβ_42_ ratio may provide good diagnostic accuracy for the differential diagnosis of AD from other disorders [77].

Keeping the above limitations in mind, biomarkers are not useful only in research, but also in clinical practice, showing both added and prognostic value [78,79]. They are not only able to confirm the presence of AD in typical cases, but they are able to identify AD in cases with atypical presentations such as primary progressive aphasia, corticobasal syndrome and posterior cortical atrophy [34,35,80,81]. Correct recognition of Alzheimer’s disease (not just amyloid pathology) helps to avoid administration of aducanumab (or any other anti-amyloid antibody) in non-AD patients, and makes possible the use of such disease-modifying treatments in clinically atypical AD patients.

The question arises as to whether amyloid PET alone is sufficient for the diagnosis of AD (especially in atypical presentations) and whether an additional tau PET would be required, with concerns regarding cost-effectiveness. CSF biomarkers are of much lower cost, but require a lumbar puncture which, being relatively invasive, is a source of concern in many patients and/or caregivers. Blood-based biomarkers, including Aβ_42_ and various forms of phospho-tau, might replace CSF and PET biomarkers in the future, since they are easily obtained, not only for initial diagnosis but also for repeated follow-up [82]; however, sensitivity and specificity to effectively discriminate AD from other dementias still has to be established [83].
